# Citral-Containing Essential Oils as Potential Tyrosinase Inhibitors: A Bio-Guided Fractionation Approach

**DOI:** 10.3390/plants10050969

**Published:** 2021-05-13

**Authors:** Francesca Capetti, Massimo Tacchini, Arianna Marengo, Cecilia Cagliero, Carlo Bicchi, Patrizia Rubiolo, Barbara Sgorbini

**Affiliations:** 1 Dipartimento di Scienza e Tecnologia del Farmaco, Università degli Studi di Torino, Via Pietro Giuria 9, I-10125 Turin, Italy; francesca.capetti@unito.it (F.C.); arianna.marengo@unito.it (A.M.); cecilia.cagliero@unito.it (C.C.); carlo.bicchi@unito.it (C.B.); patrizia.rubiolo@unito.it (P.R.); 2 Dipartimento di Scienze della vita e Biotecnologie, Università degli Studi di Ferrara, Via L. Borsari 46, I-44121 Ferrara, Italy; massimo.tacchini@unife.it

**Keywords:** tyrosinase inhibition, essential oils, citral

## Abstract

Excessive melanin production causes serious dermatological conditions as well as minor aesthetic problems (i.e., freckles and solar lentigo). The downregulation of tyrosinase is a widespread approach for the treatment of such disorders, and plant extracts have often proven to be valuable sources of tyrosinase inhibitors. Citral (a mixture of neral and geranial) is an important fragrance ingredient that has shown anti-tyrosinase potential. It is highly concentrated in the essential oils (EOs) of *Cymbopogon schoenanthus* (L.) Spreng., *Litsea cubeba* (Lour.) Pers., *Melissa officinalis* L., and *Verbena officinalis* L. However, only *L. cubeba* EO has been investigated for use as a potential skin-whitening agent. This work evaluates the in vitro tyrosinase inhibitory activity of these EOs and studies, using bio-assay oriented fractionation, whether their differing chemical compositions influence the overall EO inhibitory activities via possible synergistic, additive, and/or competitive interactions between EOs components. The inhibitory activity of *C. schoenanthus* EO and that of *M. officinalis* EOs, with negligible (+)-citronellal amounts, were in-line with their citral content. On the other hand, *L. cubeba* and *V. officinalis* EOs inhibited tyrosinase to considerably greater extents as they contained β-myrcene, which contributed to the overall EO activities. Similar observations were made for *M. officinalis* EO, which bears high (+)-citronellal content which increased citral activity.

## 1. Introduction

Tyrosinase is the key enzyme in the biosynthesis of melanin pigments in several bacteria, fungi, plants, animals, and humans. In humans, tyrosinase catalyzes the rate limiting steps in the melanin biosynthetic pathway. This biosynthesis is characterized by several enzymatic and chemical reactions that lead to melanin formation from the amino acid L-tyrosine, with tyrosinase catalyzing its hydroxylation to *o*-dopaquinone via its monophenolase and diphenolase activities. Although there are other enzymes involved in melanogenesis, only the tyrosinase-catalyzed reactions cannot occur spontaneously, whereas the remaining steps can proceed without enzyme activity at physiological pH [1]. For this reason, tyrosinase downregulation is a very widespread approach to the reduction of excessive melanin production, and the use of tyrosinase inhibitors as skin-whitening agents has demonstrated significant clinical and cosmetic prominence [2].

In the EU market, the tyrosinase inhibitors that are employed as skin-whitening agents can be grouped into two main categories: those banned under EU cosmetic regulation 1223/2009 (i.e., hydroquinone and monobenzyl ether hydroquinone) due to their severe side effects, but that are still used to treat hyperpigmentation under medical supervision; and tyrosinase inhibitors that are permitted for use in cosmetics products (i.e., arbutin, aloesin, kojic acid) [2,3]. This second group, however, is still characterized by potentially significant side-effects; clinical studies on kojic acid have indeed highlighted cases of erythema, stinging sensations, and contact eczema after application. Similarly, the European Scientific Committee on Consumer Safety has raised concerns regarding the use of arbutin as a cosmetic ingredient [2], due to the potential hydrolysis of its glyosidic bond that releases hydroquinone. There is therefore a need for novel molecule templates and/or mixtures of bioactive compounds to treat hyperpigmentation.

Plants have been valuable sources of skin-whitening agents, and three out of five of the most employed agents, both medically and cosmetically, are plant specialized metabolites (i.e., hydroquinone, β-arbutin, aloesin). To date, phenolic compounds have principally been investigated as potential tyrosinase inhibitors, and these include flavonoids (e.g., quercetin [4]), stilbenes (e.g., resveratrol [1]), phenylpropanoids (e.g., cinnamaldehyde [5] and eugenol [6]), and phenolic acids (e.g., anisic acid and benzoic acid [7]). The interest for terpenoids has been considerably lower and they have relatively been under-investigated as anti-tyrosinase agents.

Citral is among the limited number of terpenoid derivatives with anti-tyrosinase properties that have been studied. It is a mixture of two isomers, *cis*- and *trans*-3,7-dimethyl-2,6-octadienal (i.e., neral and geranial), which have been proven to block the in vitro enzymatic activity of mushroom tyrosinase [8]. In addition to its importance as odorous ingredient in beverages, foods, and cosmetics, citral has shown promising in vitro biological activities including anti-fungal, anti-bacterial, antioxidant, and anti-inflammatory effects [9,10,11]. Moreover, recent studies have highlighted that citral has potential therapeutic significance as a smooth muscle relaxer and local anesthetic, as it promotes relaxation in tracheal, uterine, and aortic smooth muscles and inhibits nerve excitability in animal models [12,13,14,15].

Citral is obtained from the essential oils (EOs) of several botanical species, including *Cymbopogon schoenanthus* (L.) Spreng., *Litsea cubeba* (Lour.) Pers., *Melissa officinalis* L., and *Verbena officinalis* L. To the best of the authors’ knowledge, only *L. cubeba* EO has been investigated for its tyrosinase inhibitory activity [16]. Therefore, this study aims to evaluate the tyrosinase inhibitory activities of *C. schoenanthus*, *L. cubeba*, *M. officinalis*, and *V. officinalis* EOs, using an in vitro colorimetric assay, to assess whether the different chemical compositions influence the overall EO inhibitory activities via any possible synergistic, additive and/or competitive interactions between their components. This study uses a bioassay-guided fractionation approach to evaluate comprehensively the EOs constituents and their enantiomers, when chiral, that contribute to the EO inhibitory activity against a mushroom source of tyrosinase, which is a good model system for the preliminary screening of tyrosinase inhibitors [17].

## 2. Results and Discussion

### 2.1. Chemical Composition and Citral Content of the Investigated Essential Oils

**Figure 1:**
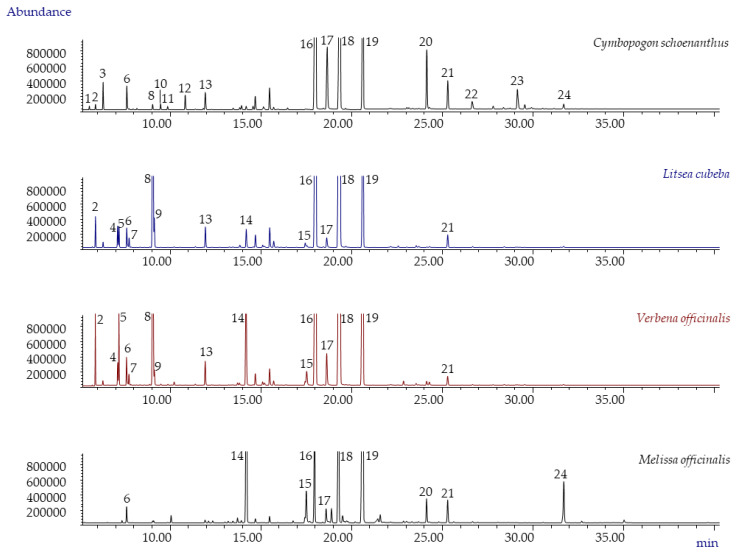
GC-MS profiles of *Cymbopogon schoenanthus* (*batch* 2020), *Litsea cubeba* (*batch* 2020), *Verbena officinalis* (*batch* 2020), and *Melissa officinalis* 1 essential oils. Legend: (1) tricyclene, (2) α-pinene, (3) camphene, (4) sabinene, (5) β-pinene, (6) 6-methyl-5-hepten-2-one, (7) β-myrcene, (8) limonene, (9) 1,8-cineole, (10) *cis*-β-ocimene, (11) *trans*-β-ocimene, (12) 4-nonanone, (13) linalool, (14) citronellal, (15) nerol, (16) neral, (17) geraniol, (18) geranial, (19) ISTD (C13), (20) geranyl acetate, (21) *trans*-β-caryophyllene, (22) *trans*-isoeugenol, (23) γ-cadinene, (24) caryophyllene oxide. For analysis conditions, see Section 3.4.

In our attempt to comprehensively characterize all of the potential EO components that contribute to the considered biological activity, the investigated EOs were analyzed by GC, with both FID and MS detection. The normalized relative percentage abundances (calculated from the absolute areas normalized to the internal standard C13 by using response factors [18,19]) of all the detected compounds were determined and used to compare EO compositions. Figure 1 reports the GC-MS profile of the investigated EOs analysed with a conventional column. Table 1 lists, for each investigated EO, the compounds that displayed a normalized percentage abundance above 0.1, while the complete EO chemical compositions are reported in the Supplementary Materials (Tables S1–S5).

All of the investigated EOs are rich in neral (*cis*-3,7-dimethyl-2,6-octadienal) and geranial (*trans*-3,7-dimethyl-2,6-octadienal), which are the most abundant compounds. The neral/ geranial ratio was very similar in all the investigated EOs and corresponded to 0.74 ± 0.05. The *C. schoenanthus* and *L. cubeba* EOs display the highest neral and geranial content, which accounts for, on average, 60% of their entire EO compositions, and which is 1.5-times greater than in *V. officinalis* EO and in the three *M. officinalis* EOs (i.e., Sample 1, 2, and 3). The additional oxygenated compounds that are common to all the EOs are 6-methyl-5-hepten-1-one, linalool and citronellal. The latter is significantly more abundant in the *M. officinalis* EO 1 than in the other investigated EOs, including the *M. officinalis* EO 2 and 3. 

The abundance of the hydrocarbon fraction varies significantly in the different EOs. *M. officinalis* EO 1 contains only *trans*-β-caryophyllene and α-humulene as sesquiterpene hydrocarbons, which account for 2.7% and 0.13% of the total EO, respectively. The *C. schoenanthus* EO presents a slightly richer hydrocarbon fraction than *M. officinalis* EO 1 (i.e., 7.0%), containing both monoterpenes (i.e., camphene, *cis*-β-ocimene, limonene, α-pinene, *trans*-β-ocimene, α-thujene) and sesquiterpenes (i.e., *trans*-β-caryophyllene, γ-cadinene, δ-cadinene, germacrene D, β-elemene) in moderate amounts. In the *L. cubeba* and *V. officinalis* EOs, the hydrocarbon fraction accounts for 20% of the total EO and limonene is the most abundant compound (i.e., 15.0 and 10.9%, respectively), followed by α-pinene, β-pinene, sabinene, *trans*-β-caryophyllene, β-myrcene, camphene, and α-copaene. Finally, *M. officinalis* EO 2 and 3 are characterized by the highest hydrocarbon fraction content (38.8% and 31.8% of the total EO, respectively). In both samples, the hydrocarbon fraction mainly contains sesquiterpenes, namely *trans*-β-caryophyllene (27.8% and 20.0%, respectively), and α-humulene (3.0% and 2.6%), and a reduced monoterpene fraction that is mainly characterized by limonene (4.2% and 3.2%, respectively). 

Three samples of *L. cubeba*, *V. officinalis*, and *C. schoenanthus* Eos produced in different years as well as three samples of *M. officinalis* EOs from distinct manufactures were investigated. GC-MS analyses of *C. schoenanthus*, *L. cubeba*, *M. officinalis*, and *V. officinalis* did not reveal significant qualitative and quantitative differences in the chemical composition of the three samples of different years of production. This may be ascribed to optimal storage conditions, i.e., in an amber-glass container at 4 °C in the dark with a negligible head space. On the other hand, GC-MS analyses showed significant differences in the abundances of citronellal and *trans*-β-caryophyllene in the three investigated *M. officinalis* EOs. Citronellal amounted to 19.6%, 0.26%, and 0.31% in the *M. officinalis* EO 1, 2, and 3, respectively. On the contrary, as previously described, *trans*-β-caryophyllene is considerably more abundant in the *M. officinalis* EOs 2 and 3 than in *M. officinalis* EO 1. These results are in agreement with the findings reported by Seidler-Lozykawska et al., who highlighted significant differences in citral, citronellal, and *trans*-β-caryophyllene abundances in the EOs obtained from 22 selected *M. officinalis* genotypes originating from European botanical gardens [20]. 

A true quantitation was performed by the external standard calibration to accurately evaluate the abundance of potential bioactive specialized compounds (i.e., neral, geranial, limonene, β-myrcene, and citronellal. Table 2 and Table 3 report the diagnostic ions (*m*/*z*) used for SIM-MS quantitation of the marker compounds under investigation together with the calibration range, the calibration curve equation, correlation values, and regression standard error of each analyte and the quantitation results, respectively. 

### 2.2. In Vitro Inhibitory Activity of the Investigated Essential Oils against Mushroom Tyrosinase

As previously described, the EOs of *C. schoenanthus*, *M. officinalis*, *L. cubeba*, and *V. officinalis* present high levels of citral, which is characterized by non-competitive inhibitory activity against a fungal source of tyrosinase [8,16,21]. This study aimed at examining the in vitro tyrosinase inhibitory activities of these EOs to explore whether their inhibitory activity can be ascribed to their citral content only, or whether there are other bioactive compounds that influence the inhibitory effects of the EOs. 

Mushroom tyrosinase was here adopted because of its high homology to human tyrosinase, its relatively low cost and ready availability, which make it a good model system for the preliminary screening of tyrosinase inhibitors [17]. The precision of the in vitro tyrosinase inhibition test was evaluated in terms of repeatability (by performing the enzymatic inhibition assay five times in the same day) and intermediate precision (by repeating the enzymatic inhibition assay five times every four weeks over a period of six months). Table 4 reports the coefficient of variation (CV) for inhibition tests carried out with kojic acid, which was used as a positive control, and with *L. cubeba* EO. Results were satisfactory as the CV never exceeded 7% for repeatability and 10% for intermediate precision. Table 4 reports the coefficient of variation for inhibition tests carried out with kojic acid, used as positive control, and with *L. cubeba* EO. Similar precision values were obtained for all the tested EOs.

Citral concentration–response curve was studied by plotting the observed inhibitory activity as a function of its concentration in the reaction mixture. All of the EOs were tested at a concentration of 166.7 μg/mL, which provided, irrespective of the EO, a resulting citral concentration within its concentration-response curve linearity range (y = 0.3956x + 1.8094, R^2^ = 0.9951, regression error: 2.08448, linearity range: 6.7–166.7 µg/mL) and did not generate solubility issues in the reaction mixture. 

**Figure 2:**
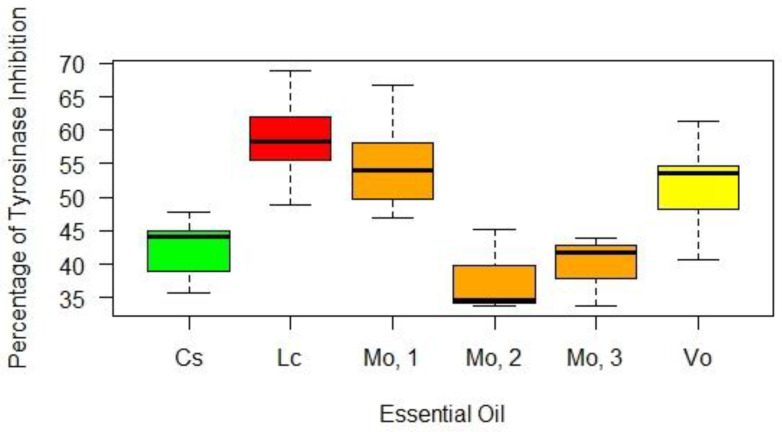
Percentage of tyrosinase inhibition for each investigated EO tested at a concentration of 166.7 µg/mL. Legend: Cs: *Cymbopogon schoenanthus (batch* 2020); Lc: *Litsea cubeba (batch* 2020); Mo,1: *Melissa officinalis* 1; Mo,2: *Melissa officinalis* 2; Mo,3: *Melissa officinalis* 3; Vo: *Verbena officinalis (bacth* 2020).

The box plot reported in Figure 2 presents the percentage of tyrosinase inhibition for each EO. For *L. cubeba*, *V. officinalis*, and *C. schoenanthus* EOs, the results reported in Figure 2 correspond to the mushroom tyrosinase inhibitory activity of the EOs of 2020 because the analysis of variance revealed no statistically significant differences among EOs of different years of production (*p* > 0.05). In regard to *L. cubeba* and *C. schoenanthus* EOs, these outcomes are in good agreement with the results obtained from the quantitative GC-MS analyses that revealed an almost identical citral amount in the EOs of different years of production. The batch 2020 of *V. officinalis* EO contains a slightly higher citral amount than the batches 2019 and 2018. However, according to citral concentration–response curve, the citral excess in batch 2020 is not sufficient to determine a statistically significant higher percentage of enzymatic inhibition considering the random error associated with the measurements. For additional details, see Figure S1 in the Supplementary Material. On the other hand, the analysis of variance (ANOVA) followed by Tukey–Kramer post-hoc test revealed that the three tested *M. officinalis* EOs, provided by distinct manufacturers, inhibited mushroom tyrosinase to different extents, which will be further described in the following paragraphs. The greatest inhibitory activities were observed for the EOs of *L. cubeba*, *M. officinalis* 1, and *V. officinalis*, which inhibited 59 ± 6%, 55 ± 7%, and 52 ± 6% of tyrosinase activity, respectively, when tested at a concentration of 166.7 μg/mL. Statistically significant (*p* < 0.05) lower activities were observed for the EOs of *C. schoenanthus* and *M. officinalis* 2 and 3 whose enzyme inhibitory activity was 42 ± 5%, 40 ± 5%, and 38 ± 6%, respectively. Table 5 provides the inhibitor concentration that halved the enzyme activity in the given experimental conditions (IC_50_) for each investigated inhibitor (i.e., EOs, single compounds, and kojic acid). All of the EOs effectively inhibited mushroom tyrosinase and displayed an inhibitory activity that was, on average, 100-times lower than that of kojic acid, which was used as the positive control. 

### 2.3. Identification of Additional Bioactive Components, Besides Citral, by Bioassay-Guided Fractionation

**Figure 3:**
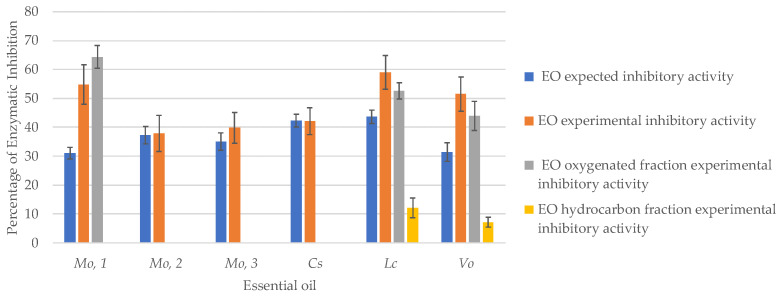
Comparison of the percentage of experimentally measured enzymatic inhibition and the enzymatic inhibition expected with citral as the only bioactive compound in the essential oils. Legend: Cs: *Cymbopogon schoenanthus (batch* 2020); Lc: *Litsea cubeba (batch* 2020); Mo,1: *Melissa officinalis* 1; Mo,2: *Melissa officinalis* 2; Mo3: *Melissa officinalis* 3; Vo: *Verbena officinalis (batch* 2020).

The histogram reported in Figure 3 compares the percentage of experimentally measured enzymatic inhibitions to the values that would be expected if neral and geranial (considered as sum, i.e., citral) were the only active compounds in the investigated EOs. These values were measured via interpolation from the citral concentration–response curve. As can be noted, *C. schoenanthus*, *M. officinalis* 2, and *M. officinalis* 3 displayed inhibitory activities that were in-line with their citral content, while *L. cubeba, M. officinalis* 1, and *V. officinalis* EOs inhibited mushroom tyrosinase to a greater extent than expected. 

A bio-guided approach was adopted to identify the additional compounds that contribute to citral activity. The oxygenated and hydrocarbon fractions of the *L. cubeba, M. officinalis* 1, and *V. officinalis* EOs were isolated by flash chromatography and individually tested for their mushroom tyrosinase inhibitory activities. The fractions phytohemical compositions are reported in the Supplementary Materials (Tables S1–S5). The isolated fractions were tested at the same concentration as their resulting concentration when testing 166.7 μg/mL of the respective EO (see Materials and Methods section at Section 3.2). Table 6 reports the concentration of neral, geranial, citronellal, limonene, and β-myrcene in the oxygenated and hydrocarbon fractions of the fractionated EOs.

As for *L. cubeba* and *V. officinalis* EOs, both the oxygenated and hydrocarbon fractions inhibited mushroom tyrosinase, although to different extents. The activities of the oxygenated fractions (53 ± 3% and 44 ± 5, respectively) account for most of the EOs anti-tyrosinase potential and were in-line with the respective citral content, suggesting that the compounds that contribute to citral activity belong to the hydrocarbon fractions. The hydrocarbon fractions of the *L. cubeba* and *V. officinalis* EOs present quite similar chemical compositions. Limonene (68.4 and 50.3%, respectively), *trans*-β-caryophyllene (12.0 and 7.8%, respectively), α-pinene (1.7 and 7.5%, respectively), β-pinene (2.5 and 12.9%, respectively), sabinene (2.7 and 3.8, respectively), and β-myrcene (2.0 and 2.4%, respectively) are the most abundant compounds in both fractions and are present in rather similar amounts, except for α-pinene and β-pinene, which prevail in the *V. officinalis* EO hydrocarbon fraction. 

The chiral recognition revealed high enantiomeric purities in favor of the (-)-configured enantiomers for *trans*-β-caryophyllene (>99% in both EOs), limonene (97 and 94% in *L. cubeba* and *V. officinalis* EO, respectively) and sabinene (87% in both EOs), while different enantiomeric excesses were observed for α-pinene ((-)-enantiomer: 38% in *L. cubeba* EO and 73% in *V. officinalis* EO) and β-pinene ((-)-enantiomer: 67%in *L. cubeba* EO and 88% in *V. officinalis* EO). In both EOs, (-)-limonene accounts for more than 50% of the entire fraction. However, although previous studies have reported an inhibitory activity against mushroom tyrosinase because of its high abundance [22,23], (-)-limonene here did not show a tyrosinase inhibitory activity. Similar results were obtained for (+)-limonene, the racemic mixture, and the compounds (-)-*trans*-β-caryophyllene, (±)-α-pinene, and (±)-β-pinene. Sabinene was not tested as it had already been proven to have negligible mushroom tyrosinase inhibitory effects [8]. In agreement with previous findings [8], β-myrcene reduced mushroom tyrosinase activity. When tested at the concentration observed in 166.7 μg/mL of *L. cubeba* and *V. officinalis* EOs_,_ β-myrcene activity bridged the gap between the EOs’ expected inhibitory effects if citral was the only active compound and the experimental results. Contrary to the observations by Matsuura et al. [8], β-myrcene proved to be a more potent mushroom tyrosinase inhibitor than citral, as its IC_50_ was almost ten times lower (13.3 μg/mL vs. 121.8 μg/mL). This difference may be ascribed to the different substrates used; Matsuura et al. investigated mushroom tyrosinase diphenolase activity only, as they used L-DOPA as the substrate, whereas, in this study, L-tyrosine was used. The current findings suggest that β-myrcene may be more effective at inhibiting mushroom tyrosinase monophenolase activity than the diphenolase one. 

The *M. officinalis* EO 1 displays a small hydrocarbon fraction that accounts for less than 3% of the total, and has no tyrosinase inhibitory activity. However, the *M. officinalis* EO 1 oxygenated fraction inhibited mushroom tyrosinase to a greater extent than would be expected from its citral content (Figure 3). This fraction contains significant amounts of citronellal in addition to neral and geranial and the chiral analysis revealed a high enantiomeric purity of citronellal in favor of the (+) enantiomer (98.3%). When tested independently, at a concentration of 166.7 μg/mL, (+)-citronellal inhibited mushroom tyrosinase to a negligible extent, although its activity was significantly enhanced when tested in combination with citral. These results may explain the differences observed in the percentages of mushroom tyrosinase inhibition in the various *M. officinalis* EOs. *M. officinalis* EO 2 and 3 present very low citronellal contents, which may be the reason why their inhibitory activities are significantly lower than that of *M. officinalis* EO 1. 

## 3. Materials and Methods

### 3.1. Reagents

Dimethyl sulfoxide (DMSO), mushroom tyrosinase from *Agaricus bisporus* (J.E. Lange) Imbach, L-tyrosine, kojic acid, citral, citronellal, β-myrcene, (+)-limonene, (-)-limonene, (±)-limonene, (±)-α, and β pinene were purchased from Merck Life Science S.r.l. (Milan, Italy). *Litsea cubeba*, *Verbena officinalis*, and *Cymbopogon schoenanthus* EOs were supplied by Erboristeria Magentina S.r.l. (Poirino, Italy). Three batches of different years (i.e., 2020, 2019, 2018) were tested for each. Three samples of *Melissa officinalis* EOs were investigated; one was provided by Agronatura (Spigno Monferrato, Alessandria), one by Erboristeria Magentina S.r.l., while the last was purchased from a local shop and was from Specchiasol S.r.l. (Bussolengo, Italy). In the text, the authors refer to the different EOs of *Melissa officinalis* as *M. officinalis EOs* 1, 2, and 3, respectively. The provided EOs were obtained following the procedures described in the European Pharmacopoeia [24]. *Melissa officinalis* and *Verbena officinalis* EOs were produced by hydrodistillation from the leaves and plants aerial parts, respectively; similarly, *Litsea cubeba* and *Cymbopogon schoenanthus* EOs were obtained by steam distillation of the fresh fruits and fresh aerial parts, respectively. Each EO was individually analyzed by GC-MS as soon as it was purchased/provided by the corresponding manufacturer, every storage year, and just before the study of its mushroom tyrosinase inhibitory activity.

### 3.2. In Vitro Tyrosinase Inhibitory Assay 

The tyrosinase inhibitory activities of the EOs and isolated compounds were investigated in vitro using a colorimetric readout-based enzyme assay optimized by Zengh et al. [25], with slight modifications. The tyrosinase inhibitory activities of EOs, as well as their respective hydrocarbon and oxygenated fractions and pure compounds were investigated in vitro using a colorimetric readout-based enzyme assay that was optimized by Zengh et al. [25], with slight modifications: the assay was carried out at room temperature and tyrosinase inhibition was measured considering control and sample absorbance after 6 min of incubation, rather than after 20 min, so as to operate under the linear portion of the enzymatic reaction, which provides more accurate inhibition results [26,27]. Mushroom tyrosinase from *Agaricus bisporus* (J.E. Lange) Imbach was selected for this study. L-Tyrosine was used as the substrate, meaning that the overall tyrosinase inhibitory activity was investigated without distinguishing between tyrosinase monophenolase and diphenolase activity. Photometric measurements at 475 nm were performed on a Thermo spectronic Genesys 6 and kojic acid was used as the positive control inhibitor. The solutions of the investigated potential inhibitors (EOs, EO isolated fractions, EO individual compounds, and kojic acid) were prepared in DMSO. Table 7 reports the tested concentrations for each investigated potential inhibitor. The mushroom tyrosinase solution 200 U/mL (27.9 μg/mL) was prepared in sodium phosphate buffer (pH 6.8) and aliquots of 9 mL were stored at −18 °C and thawed just before the experiments. Tyrosine solution 0.1 mg/mL was prepared in sodium phosphate buffer (pH 6.8) and renewed daily. The reaction mixture components were placed in the vial in the following order: 1 mL of mushroom tyrosinase solution 200 U/mL; 1 mL of sodium phosphate buffer solution; 10 μL of EO/single compound/kojic acid solution; and, finally, 1 mL of tyrosine solution 0.1 mg/mL. The final DMSO percentage in the reaction mixture was 0.3%. The assay was performed in a sealed 4 mL vial to avoid the loss of any EO components into the surrounding environment and to minimize their release into the head space above the reaction mixture. The reaction mixture was incubated in a thermostatic water bath at 25 °C for 6 min. Subsequently, the absorbance at 475 nm was registered, as this wavelength allows the identification of dopachrome. The absorbance corresponding to 100% of tyrosinase activity was measured by replacing the EOs/individual compound/kojic acid solution with 10 μL of pure DMSO. Blank solutions were prepared as follows: 2 mL of sodium phosphate buffer solution, 10 μL of EO/individual compound/kojic acid/DMSO solution, and 1 mL of tyrosine solution 0.1 mg/mL. The percentage of tyrosinase inhibition was measured according to the equation below:

% Inhibition = ΔA (Control) − ΔA (Sample) / ΔA (Control) × 100,

ΔA (Control) or (Sample) = A_475_ (Control) or (Sample) − A_475_ (Control Blank) or (Sample Blank).

#### Determination of Concentration–Response Curve and IC_50_ for Inhibitors 

The concentration-response curve for each inhibitor was determined by plotting the inhibitory activity as a function of inhibitor concentration in the reaction mixture. IC_50_ values for the inhibitors were obtained by interpolation from the concentration–response curve. 

### 3.3. Flash Column Chromatography

EO fractionation was carried out on a flash column chromatography system PuriFlash 450 by Sepachrom (Rho, Milan, Italy), equipped with both UV and ELSD detectors. The amount of EO fractionated: 900.0 mg. Stationary phase: spherical silica gel particles, 50 μm, 25 mg (Purezza^®^-Sphera Cartridge Stationary) was from Sepachrom; mobile phase: petrolether (A) and ethyl acetate (B); flow-rate 25 mL/min. Linear gradient elution was adopted from 100% of A to 80% of A and 20% of B over 20 min. 

### 3.4. Analysis Conditions

The EOs solutions and those of their respective fractions were prepared in cyclohexane at a concentration of 5.0 mg/mL and analyzed by GC-MS. Citral, citronellal, β-myrcene, and limonene were quantified in each EO and the corresponding isolated fractions using the external standard calibration method. Suitable calibration levels were prepared in cyclohexane and analyzed by GC-MS. Tridecane (C13) 1.0 mg/mL was used as the internal standard to normalize the analyte signals. Table 2 summarizes the considered concentration range for each quantified compound. 

GC-MS analyses were carried out using a Gerstel MPS-2 multipurpose sampler (Mülheim an der Ruhr, Germany) installed on an Agilent 6890 N GC coupled to a 5975 MSD and equipped with a ChemStation Version E.02.02.1431 data processing system (Agilent Technologies, Santa Clara, CA, USA). GC conditions: injector temperature: 250 °C; injection mode: split; ratio: 1/20; carrier gas: helium; constant flow rate: 1 mL/min; columns: Mega 5 (95% polydimethylsiloxane, 5% phenyl) df 0.25 µm, dc 0.25 mm, length 25 m, from MEGA (Legnano, Italy). Temperature program: 50 °C//3 °C/min//180 °C//10 °C/min//250 °C (5 min). MSD conditions: MS operated in EI mode (70 eV); scan range: 35 to 350 amu; dwell time 40 ms; ion source temperature: 230 °C; quadrupole temperature: 150 °C; transfer-line temperature: 280 °C. EO markers were identified by comparing both their linear retention indices (ITs), calculated versus a C9-C25 hydrocarbon mixture, and their mass spectra either against those of authentic samples, or from commercially available mass spectral libraries (Adams, 2007). EO chiral analyses were performed by adopting the same analysis conditions on a 2,3-di-O-methyl-6-O-t-butyldimethylsilyl-β-CD (2,3DM6TBDMS-β-CD) df 0.25 µm, dc 0.25 mm, length 25 m from MEGA. Temperature programs: 40 °C (1 min)//2 °C/min//220 °C (5 min).

GC-FID analyses were carried out on the same instrument. GC conditions: injector temperature: 250 °C; injection mode: split; ratio: 1/20; carrier gas: hydrogen; flow rate: 1 mL/min. Temperature programs: 40 °C (1 min)//2 °C/min//220 °C (5 min). 

## 4. Conclusions

The purposes of this investigation were (1) to examine comprehensively the in vitro mushroom tyrosinase inhibitory activities of the *Cymbopogon schoenanthus*, *Litsea cubeba*, *Melissa officinalis*, and *Verbena officinalis* EOs and (2) to determine whether their biological activity is ascribed to their citral content only or if there are additional bioactive monoterpenes that contribute to the investigated biological activity by using a bioassay-guided fractionation approach. This study has identified that in *L. cubeba* and *V. officinalis* EOs, the β-myrcene contributes to the EOs inhibitory activities despite its little amount and it has been shown to have a greater inhibitory power to citral. The second major finding was that (+)-citronellal enhanced citral mushroom tyrosinase inhibitory power, potentially via synergistic interaction as it displayed no activity on its own. The latter finding explained why in *M. officinalis* EOs that bear negligible (+)-citronellal amounts, the inhibitory activities were in-line with their citral content while the contrary was true for the *M. officinalis* EO with relatively high (+)-citronellal abundance.

Even though further studies are still required to accurately define the type of interactions that occur in between β-myrcene and citral and in between citronellal and citral, and to assess the inhibitory activities of these EOs and individual compounds on human tyrosinase, the results of this study may help to rationally design mixtures of EOs or enriched EOs that improve their biological efficacy and increase their potential as adjuvants in the treatment of hyperpigmentation. 

## Figures and Tables

**Table 1:** Normalized percentage abundance of the compounds identified in the essential oils under investigation. For complete compositions, see Supplementary Materials Table S1–S5.

Compound			*C. schoenanthus*		*L. cubeba*		*V. officinalis*		*M. officinalis* *_1_*		*M. officinalis* *_2_*		*M. officinalis* *_3_*	
	I^t^s_exp_	I^t^s_lit_	Norm. Rel. % Abundance *	CV	Norm. Rel. % Abundance *	CV	Norm. Rel. % Abundance *	CV	Norm. Rel. % Abundance	CV	Norm. Rel. % Abundance	CV	Norm. Rel. % Abundance	CV
Tricyclene	926	930											0.11	2.5
α-Thujene	930	931	0.15	11.7										
α-Pinene	941	939	0.22	8.4	1.3	0.6	3.7	1.2			0.43	1.2	0.40	3.9
Camphene	954	953	1.2	4.2	0.26	0.4	0.22	1.9			0.31	0.7	0.95	2.6
Sabinene	976	976			0.97	2.6	1.1	0.2			0.17	8.4	0.13	4.8
β-Pinene	978	980			1.0	3.1	4.0	0.2			0.55	12.5	1.0	3.0
1-Octen-3-ol	982	978							0.21	4.4				
6-Methyl-5-hepten-1-one	989	989	1.2	3.2	1.0	5.2	1.5	1.0	1.4	0.5	0.51	5.8	0.87	2.6
β-Myrcene	992	991			0.47	5.5	0.57	0.8			0.14	4.3		
*p*-Cymene	1026	1024									0.11	0.2		
Limonene	1029	1031	0.29	5.9	15.0	0.1	10.9	5.4			4.2	0.2	3.7	2.0
1,8-Cineole	1030	1033			1.5	0.1	0.78	6.0			0.91	0.1	0.34	2.5
*cis*-β-Ocimene	1040	1040	0.31	5.0										
*trans*-β-Ocimene	1050	1050	0.19	0.6										
γ-Terpinene	1059	1062					0.20	1.4			0.29	2.0		
α-Terpinolene	1086	1088												
Linalool	1098	1098	1.1	0.7	1.1	9.7	1.5	7.8	0.32	1.6	1.2	2.6	0.95	4.8
Nonal	1098	1103							0.17	1.7				
*cis*-Rose oxide	1109	1111							0.20	0.3				
*trans*-Rose oxide	1126	1127							0.10	0.8				
Isopulegol	1144	1146					0.14	3.1	0.52	2.8				
Citronellal	1155	1153	0.22	9.7	1.1	10.4	5.2	1.3	19.6	0.4	0.26	5.8	0.31	1.6
Borneol	1163	1165	0.24	2.3										
4-Terpineol	1175	1177			0.17	7.9	0.25	0.2					0.20	5.5
α-terpineol	1188	1189	0.18	1.0	0.40	9.5	0.32	8.7			0.22	1.5		
Nerol	1229	1228			0.32	10.6	0.25	4.5	0.45	3.9				
*trans*-β-Citronellol	1231	1228			0.13	3.6	1.2	0.5	4.1	0.7			0.11	1.8
Neral	1243	1240	32.0	0.2	30.8	0.3	27.5	0.1	19.7	0.1	21.4	0.8	16.5	0.7
Piperitone	1252	1252							0.10	2.8			0.17	1.4
Geraniol	1257	1255	5.16	6.3	0.78	0.8	2.4	0.2	1.7	2.7	1.6	0.5	3.3	1.4
Methyl citronellate	1263	1261							1.6	1.8				
Geranial	1274	1270	41.8	1.1	39.4	1.8	33.2	0.6	29.6	0.2	28.8	0.2	26.5	0.2
Citronellyl formate	1275	1277							1.0	0.2			0.66	0.6
α-Terpinyl acetate	1348	1350			0.11	1.8								
α-Cubebene	1351	1347									0.33	0.5	0.34	0.2
Methyl geranate	1323	1324							0.86	1.6				
Citronellyl acetate	1355	1354					0.30	0.5	0.18	4.0				
Neryl acetate	1365	1366							0.13	0.53			0.26	2.3
α-Copaene	1371	1372			0.13	9.8	0.13	4.1			0.79	0.4	0.81	0.3
Geranyl acetate	1384	1383	4.2	0.6			0.29	1.0	2.5	0.2	0.92	0.4	1.6	0.2
β-Elemene	1388	1391	0.13	3.9			0.25	0.3			0.09	3.6	0.12	0.1
*trans*-β-Caryophyllene	1414	1418	2.1	1.3	0.93	0.1	0.69	2.8	2.6	1.4	27.8	1.0	20.0	0.5
*trans*-Isoeugenol	1447	1450	0.71	4.4										
α-Humulene	1454	1447							0.13	7.0	3.0	0.3	2.6	0.7
Germacrene D	1475	1480	0.21	3.2										
γ-Cadinene	1508	1513	1.8	2.4							0.59	0.2	0.99	0.9
δ-Cadinene	1519	1524	0.32	1.2							0.52	2.0	0.81	2.3
Caryophyllene oxide	1575	1580	0.43	3.9	0.11	0.7			5.7	1.7	1.6	1.7	8.5	1.2

* Average values which were derived from the analyses of three EOs obtained from the same botanical species but of different years of production. CV: Coefficient of Variation = (Standard Deviation/Mean) × 100.

**Table 2:** Diagnostic ions (*m*/*z*) used for SIM-MS quantitation of selected marker compounds that characterize the investigated essential oils together with the calibration range, the calibration curve equation, correlation values, and regression standard error.

Compound	Diagnostic Ion	Calibration Range (mg/mL)	Calibration Curve Equation	Correlation Values	Regression Standard Error
Neral	69	0.39–1.95	y = 0.4548x + 0.0412	0.9983	0.01543
Geranial	69	0.61–3.05	y = 0.7701x + 0.1207	0.9964	0.05848
Citral		1.00–5.00	y = 0.7067x + 0.1034	0.9956	0.09788
Limonene	68	0.10–2.50	y = 0.6003x + 0.0828	0.9910	0.07348
β-Myrcene	93	0.01–0.08	y = 1.3304x − 0.0023	0.9994	0.001033
Citronellal	69	0.08–4.08	y = 0.5325x − 0.020	0.9999	0.01427
Citronellal	69	0.01–0.08	y = 0.4685x − 0.0083	1.0000	0.0004276

**Table 3:** Absolute concentrations of potentially bioactive components in the investigated essential oils.

Essential Oil	Batch	[β-Myrcene] (g/100 g)	CV	[Limonene] (g/100 g)	CV	[Citronellal] (g/100 g)	CV	[Neral] (g/100 g)	CV	[Geranial] (g/100 g)	CV	[Citral] (g/100 g)	CV
*L. cubeba*	2020	0.4	5.9	14.7	1.1	1.2	0.1	24.5	2.9	34.3	3.8	59.4	3.5
	2019	0.4	7.2	11.3	1.1	0.9	0.2	25.7	2.0	37.9	2.6	64.6	2.4
	2018	0.3	4.8	8.8	13.4	1.7	1.0	27.7	3.5	37.5	3.6	65.6	3.5
*C. schoenanthus*	2020	0.1	8.8	2.1	3.6	0.4	0.3	25.9	1.0	37.1	1.4	63.8	1.3
	2019	0.1	3.8	2.2	3.2	0.5	1.0	23.9	2.3	34.1	1.4	58.7	1.7
	2018	0.1	7.5	2.4	1.5	0.4	0.7	26.7	2.7	37.8	0.9	64.8	1.4
*V. officinalis*	2020	0.4	5.0	10.3	2.6	5.3	0.9	21.6	3.5	28.8	3.0	50.6	3.2
	2019	0.5	1.2	16.7	4.9	4.7	2.0	16.5	3.2	24.1	2.4	41.2	2.3
	2018	0.5	3.8	15.5	4.4	5.1	1.7	16.8	4.9	24.9	3.6	42.4	4.0
*M. officinalis*	1	0.0	8.9	0.0	-	0.4	4.5	15.5	0.3	22.4	0.0	36.0	0.1
	2	0.1	5.2	4.3	3.1	0.4	7.7	18.4	1.2	27.7	0.4	46.9	0.5
	3	0.1	9.0	3.3	2.6	15.9	3.5	18.0	0.4	28.2	0.4	44.0	0.2

**Table 4:** Data precision expressed as CV for both repeatability (n = 5) and intermediate precision (n = 6). * Values represent the average of three assays.

	Repeatability (n = 3)		Intermediate Precision	
	% Inhibition	CV	% Inhibition *	CV
***Kojic acid*** **(1.7 µg/mL)**	64	6	59	8
	58		69	
	61		67	
	66		58	
	64		70	
			66	
***Litsea cubeba*** **EO** **(166.7 µg/mL)**	57	7	51	11
	58		59	
	55		60	
	62		56	
	65		67	
			57	

**Table 5:** IC_50_ values of each investigated essential oil and of some bioactive components together with their relative standard deviation value.

Inhibitor	IC_50_ (μg/mL)
Kojic acid	1.0 ± 0.4
Citral	121.8 ± 13.7
β-Myrcene	13.3 ± 3.1
*C. schoenanthus* EO	216.7 ± 18.3
*L. cubeba* EO	125.0 ± 16.5
*M. officinalis* EO 1	152.2 ± 21.1
*M. officinalis* EO 2	220.1 ± 27.7
*M. officinalis* EO 3	209.2 ± 22.5
*V. officinalis* EO	167.0 ± 19.1

**Table 6:** Concentration of selected bioactive compounds in the oxygenated and hydrocarbon fractions of the fractionated essential oils.

	[β-Mycene] (g/100 g)	CV	[Limonene] (g/100 g)	CV	[Citronellal] (g/100 g)	CV	[Neral] (g/100 g)	CV	[Geranial] (g/100 g)	CV	[Citral] (g/100 g)	CV
*L. cubeba* hydrocarbon fraction	1.4	0.3	59.4	6.0								
*L. cubeba* oxygenated fraction					1.0	1.5	42.2	0.1	53.2	0.5	94.8	0.3
*V. officinalis* hydrocarbon fraction	1.8	0.2	47.2	0.8								
*V. officinalis* oxygenated fraction					2.0	2.8	35.1	0.6	42.3	0.2	76.9	0.3
*M. officinalis*, 1 hydrocarbon fraction												
*M. officinalis*, 1 oxygenated fraction					14.1	0.4	18.6	0.3	26.7	0.4	44.8	0.4

**Table 7:** Tested concentrations for investigated essential oils and for both the relative isolated hydrocarbon and oxygenated fractions.

Tested Sample	[Stock Solution] (mg/mL)	[Sample] _Reaction Mixture_ (μg/mL)
*L. cubeba* EO	5.0–50.0	16.7–166.7
*L. cubeba* EO oxygenated fraction	40.0	133.3
*L. cubeba* EO hydrocarbon fraction	10.0	33.3
*V. officinalis* EO	5.0–50.0	16.7–166.7
*V. officinalis* EO oxygenated fraction	40.0	133.3
*V. officinalis* EO hydrocarbon fraction	10.0	33.3
*C. schoenanthus* EO	5.0–50.0	16.7–166.7
*M. officinalis* EO 1	5.0–50.0	166.7
*M. officinalis* EO 1 oxygenated fraction	48.0	160.0
*M. officinalis* EO 1 hydrocarbon fraction	2.0	6.7
*M. officinalis* EO 2	5.0–50.0	16.7–166.7
*M. officinalis* EO 3	5.0–50.0	16.7–166.7
Citral	3.0–50.0	10–166.7
(+)-Citronellal	10.0, 50.0	33.3, 166.7
Citral + (+)-Citronellal	20.0 + 10.0	66.7 + 33.3
β-Myrcene	0.1–10.0	0.3–33.3
(−)-*trans*-β-caryophyllene	20.0	66.7
(+)-Limonene	10.0	33.3
(−)-Limonene	10.0	33.3
(±)-Limonene	10.0	33.3
(±)-α-Pinene	2.0	6.7
(±)-β-Pinene	2.0	6.7
kojic acid	0.02–0.2	0.067–0.67

## Data Availability

All figures and tables in this manuscript are original and unpublished anywhere else.

## Supplementary Materials

The following are available online at https://www.mdpi.com/article/10.3390/plants10050969/s1: Tables S1–S5: total chemical composition (expressed as normalized relative percentage area of each identified compound of the investigated essential oils).

## Abbreviations

EO	Essential Oil
GC-MS	Gas Chromatography-Mass Spectrometry
FID	Flame Ionization Detector
SIM	Single Ion Monitoring
CV	Coefficient of Variation
