# Supplementary material for: Citral-Containing Essential Oils as Potential Tyrosinase Inhibitors: A Bio-Guided Fractionation Approach
Source: Plants (Basel). 2021 May 13;10(5):969. doi: 10.3390/plants10050969 (PMC8152484; doi:10.3390/plants10050969)
Supplement: Supplementary file 1 [file plants-10-00969-s001.zip › plants-1204612-supplementary.pdf]

plants

Article

Citral-Containing Essential Oils as Potential Tyrosinase Inhibitors: A Bio-Guided Fractionation Approach

Francesca Capetti [1,†], Massimo Tacchini [2,†], Arianna Marengo [1], Cecilia Cagliero [1], Carlo Bicchi [1], Patrizia Rubiolo [1] and Barbara Sgorbini [1,*]

[1] Dipartimento di Scienza e Tecnologia del Farmaco, Università degli Studi di Torino, Via Pietro Giuria 9, I-10125 Turin, Italy; francesca.capetti@unito.it (F.C.); arianna.marengo@unito.it (A.M.); cecilia.cagliero@unito.it (C.C.); carlo.bicchi@unito.it (C.B.); patrizia.rubiolo@unito.it (P.R.)
[2] Dipartimento di Scienze della vita e Biotecnologie, Università degli Studi di Ferrara, Via L. Borsari 46, I-44121 Ferrara, Italy; massimo.tacchini@unife.it
* Correspondence: barbara.sgorbini@unito.it; Tel.: +39-011-670-7135
† These authors contributed equally to the work.

Supplementary Materials

Table S1. Normalized relative percentage abundance of the compounds identified in the essential oil of Cymbopogon schoenanthus.

	C. schoenanthus			
**Compound**	I^t_{Sexp}	I^t_{Slit}	Norm. Rel. % Abundance	RSD%
α-Thujene	930	931	0.15	11.7
α-Pinene	941	939	0.22	8.4
Camphene	954	953	1.2	4.2
6-methyl-5-hepten-1-one	989	989	1.2	3.2
β-Myrcene	989	991	0.06	2.8
Limonene	1029	1031	0.29	5.9
cis-β-Ocimene	1040	1040	0.31	5.0
trans-β-Ocimene	1050	1050	0.19	0.6
α-terpinolene	1086	1088	0.06	1.3
Linalool	1098	1098	1.1	0.7
Citronellal	1155	1153	0.22	9.7
Borneol	1163	1165	0.24	2.1
α-terpineol	1188	1189	0.18	1.0
Nerol	1229	1228	0.05	20.1
β-Citronellol	1231	1228	0.05	22.0
Neral	1243	1240	32.0	0.2
Piperitone	1252	1254	0.07	7.9
Geraniol	1257	1255	5.2	6.3
Geranial	1274	1270	41.8	1.1
Geranyl acetate	1384	1383	4.2	0.6
β-Elemene	1388	1391	0.13	3.9
trans-β-Caryophyllene	1414	1418	2.1	1.3
trans-Isoeugenol	1447	1450	0.71	4.4
Germacrene D	1475	1480	0.21	3.2
γ-Cadinene	1508	1513	1.8	2.4
δ-Cadinene	1519	1524	0.32	1.2
Caryophyllene oxide	1575	1580	0.43	3.9

Table S2. Normalized relative percentage abundance of the compounds identified in the essential oils of Melissa officinalis EO 2 and 3 (- : not detected).

			M. officinalis 2		*M. officinalis* 3	
**Compound**	I^t_{Sexp}	I^t_{Slit}	Norm. Rel. % Abundance	RSD%	Norm. Rel. % Abundance	RSD%
Tricyclene	930	926	0.03	2.1	0.11	2.5
α-Thujene	936	931	0.02	6.7	-	
α-Pinene	941	939	0.43	1.2	0.40	3.9
Camphene	954	953	0.31	0.7	0.95	2.6
Sabinene	976	976	0.17	8.4	0.13	4.8
β-Pinene	978	980	0.55	12.5	1.0	3.0
6-methyl-5-hepten2-one	989	985	0.51	5.8	0.87	2.6
β-Myrcene	992	991	0.14	4.3	0.06	13.9
p-Cymene	1024	1026	0.11	0.2	0.09	1.6
Limonene	1028	1031	4.2	0.2	3.7	2.1
1,8-Cineole	1030	1033	0.91	0.1	0.34	2.5
cis-β-Ocimene	1040	1040	0.04	0.6	-	
trans-β-Ocimene	1050	1050	0.05	4.2	-	
γ-Terpinene	1059	1062	0.29	2.1	-	
α-Terpinolene	1086	1088	0.05	12.3	-	
Linalool	1098	1098	1.2	2.6	0.95	4.8
Citronellal	1155	1153	0.26	5.8	0.31	1.6
Borneol	1163	1165	0.06	9.5	-	
4-Terpineol	1175	1177	0.03	29.3	0.20	5.5
α-Terpineol	1188	1189	0.22	1.5	-	
Nerol	1229	1229	0.08	5.2	0.06	0.5
β-Citronellol	1230	1228	0.08	1.8	0.11	1.8
Neral	1242	1240	21.4	0.8	16.5	0.7
Piperitone	1252	1252	0.06	1.0	0.17	1.4
Geraniol	1257	1255	1.64	0.5	3.3	1.4
Geranial	1273	1270	28.8	0.2	26.5	0.2
Citronellyl formate	1277	1275			0.66	0.6
α-Cubebene	1347	1351	0.33	0.5	0.34	0.2
Neryl acetate	1366	1365			0.26	2.3
α-Copaene	1371	1371	0.79	0.4	0.81	0.3
Geranyl acetate	1385	1383	0.92	0.4	1.63	0.2
β- Elemene	1388	1391	0.09	3.6	0.12	0.2
trans-β-Caryophyllene	1413	1418	27.8	1.0	20.09	0.5
α-Humulene	1447	1454	3.0	0.3	2.6	0.7
Germacrene D	1475	1480	0.06	5.5	-	
trans-γ-Cadinene	1507	1511	0.59	0.2	0.99	0.9
δ-Cadinene	1518	1524	0.52	2.0	0.81	2.3
Caryophyllene Oxide	1575	1580	1.6	1.7	8.5	1.2

Table S3. Normalized relative percentage abundance of the compounds identified in the EO of Litsea cubeba and in its hydrocarbon and oxygenated fractions (tr : trace; - : not detected).

			L. cubeba		*Hydrocarbon fraction*		*Oxygenated fraction*	
**Compound**	I^t_{Sexp}	I^t_{Slit}	Norm. Rel. % Abundance	RSD%	Norm. Rel. % Abundance	RSD%	Norm. Rel. % Abundance	RSD%
α-Thujene	936	931	0.03	11.7	0.04	6.2	-	-
α-Pinene	941	939	1.3	0.7	1.7	5.8	-	-
Camphene	954	953	0.26	0.4	0.41	2.4	-	-
Sabinene	976	976	0.97	2.6	2.7	0.4	-	-
β-Pinene	978	980	1.0	3.1	2. 5	4.2	-	-
6-methyl-5-hepten-2-one	989	985	1.0	5.2	-	-	0.90	1.0
β-Myrcene	992	991	0.47	5.5	2.0	3.1	-	-
α-Phellandrene	1002	1005	tr	-	0.05	1.5	-	-
δ-3-Carene	1008	1011	tr	-	0.14	2.5	-	-
α-Terpinene	1015	1018	tr	-	0.08	4.8	-	-
p-Cymene	1024	1026	tr	-	0.20	2.2	-	-
Limonene	1028	1031	15.0	0.1	68.4	2.1	-	-
cis-β-Ocimene	1040	1040	tr	-	0.10	3.3	-	-
trans-β-Ocimene	1050	1050	tr	-	0.15	0.7	-	-
1,8-Cineole	1030	1033	1.5	0.1	-	-	0.04	10.3
γ-Terpinene	1059	1062	0.05	19.5	0.34	0.9	-	-
α-Terpinolene	1086	1089	0.06	8.3	0.52	5.6	-	-
Linalool	1098	1098	1.1	9.7	-	-	1.5	0.5
Perillene	1099	1099	Tr	-	0.08	2.4	-	-
Citronellal	1154	1153	1.08	10.4	-	-	1.06	3.3
Borneol	1163	1165	0.04	16.6	-	-	0.06	10.0
4-Terpineol	1175	1177	0.17	7.9	-	-	0.20	4.1
α-Terpineol	1188	1189	0.40	9.5	-	-	0.54	6.5
Nerol	1229	1228	0.32	10.6	-	-	0.43	2.1
trans-β-Citronellol	1231	1228	0.13	3.6	-	-	0.16	
Neral	1243	1240	30.81	0.3	-	-	37.5	6.7
Piperitone	1252	1252	0.06	21.2	-	-	0.06	13.0
Geraniol	1257	1255	0.78	0.8	-	-	0.98	3.0
Geranial	1273	1270	39.36	1.8	-	-	48.4	6.4
α-Terpinyl acetate	1348	1350	0.11	1.8	-	-	0.07	
α-Copaene	1371	1372	0.13	9.8	1.63	3.9	-	-
Geranyl acetate	1384	1383	0.04	4.4			2.0	13.0
β-Elemene	1388	1391	0.06	13.3	0.78	4.1	-	-
trans-β-Caryophyllene	1412	1418	0.93	0.1	12.0	4.0	-	-
α-*trans*-bergamotene	1433	1436	tr	-	0.12	9.5	-	-
α-Humulene	1447	1454	0.07	0.4	1.2	8.4	-	-
allo-Aromadendrene	1454	1461	tr	-	0.09	4.7	-	-
trans-β-Farnesene	1457	1458	tr	-	0.35	3.2	-	-
Bicyclogermacrene	1490	1495	0.07	9.3	1.1	2.5	-	-
Germacrene A	1497	1503	tr	-	0.17	5.4	-	-
β-Bisabolene	1505	1509	tr	-	0.52	2.5	-	-
δ-Cadinene	1519	1524	tr	-	0.33	4.0	-	-

Compound	I^t_{Sexp}	I^t_{Slit}	*L. cubeba* Norm. Rel. % Abundance	RSD%	*Hydrocarbon fraction* Norm. Rel. % Abundance	RSD%	*Oxygenated fraction* Norm. Rel. % Abundance	RSD%
Caryophyllene oxide	1575	1580	0.11	0.7	-	-	0.64	6.4

Table S4. Normalized percentage abundance of the compounds identified in the EO of Verbena officinalis and in its hydrocarbon and oxygenated fractions (tr : trace; - : not detected).

Compound	I^t_{Sexp}	I^t_{Slit}	*V. officinalis* Norm. Rel. % Abundance	RSD%	*Hydrocarbon fraction* Norm. Rel. % Abundance	RSD%	*Oxygenated fraction* Norm. Rel. % Abundance	RSD%
α-Thujene	936	931	0.05	1.3	0.13	8.5	-	-
α-Pinene	941	939	3.7	1.2	7.5	6.9	-	-
Camphene	954	953	0.22	1.9	0.57	2.9	-	-
Sabinene	976	976	1.1	0.2	3.82	9.8	-	-
β-Pinene	978	980	4.0	0.2	12.9	11.0	-	-
6-methyl-5-hepten-2-one	989	985	1.5	1.0	-	-	0.62	6.3
β-Myrcene	992	991	0.57	0.8	2.4	0.6	-	-
δ-3-Carene	1008	1011	0.04	0.5	0.17	1.4	-	-
α-Terpinene	1015	1018	tr	-	0.13	1.5	-	-
o-Cymene	1022	1022	tr	-	0.06	2.8	-	-
p-Cymene	1024	1026	0.06	1.5	0.38	5.1	-	-
Limonene	1028	1031	10.9	5.4	50.3	4.2	-	-
1,8-Cineole	1030	1033	0.78	6.0	-	-	0.26	5.9
cis-β-Ocimene	1040	1040	0.07	6.0	0.41	11.2	-	-
trans-β-Ocimene	1050	1050	0.06	5.5	0.43	13.1	-	-
γ-Terpinene	1059	1062	0.20	1.4	1.1	9.4	-	-
α-Terpinolene	1086	1088	0.07	8.4	0.54	13.6	-	-
Linalool	1098	1098	1.5	7.8	-	-	1.9	2.2
Perillene	1099	1099	tr	-	0.15	9.7	-	-
Isopulegol	1144	1146	0.14	3.1	-	-	0.17	5.4
Citronellal	1155	1153	5.2	1.3	-	-	3.4	16.4
Borneol	1163	1165	0.04	1.3	-	-	0.05	2.8
4-Terpineol	1175	1177	0.25	0.2	-	-	0.31	3.4
α-Terpineol	1188	1189	0.32	8.7	-	-	0.48	1.4
Nerol	1229	1229	0.25	4.5	-	-	0.32	4.7
β-Citronellol	1231	1229	1.2	0.5	-	-	1.7	4.8
Neral	1243	1240	27.5	0.1	-	-	37.4	0.1
Piperitone	1252	1252	0.05	7.9	-	-	0.09	1.6
Geraniol	1257	1255	2.4	0.2	-	-	3.47	1.9
Geraniale	1273	1270	33.2	0.6	-	-	46.1	1.8
Citronellyl acetate	1355	1354	0.30	0.5	-	-	0.37	8.2
α-Cubebene	1347	1351	tr	-	0.12	9.5	-	-
α-Copaene	1371	1371	0.13	4.1	1.2	5.8	-	-
β-Bourbonene	1379	1384	tr	-	0.11	5.6	-	-
Geranyl acetate	1384	1383	0.29	1.0	-	-	0.39	3.3
β-Elemene	1388	1391	0.25	0.3	2.2	5.8	-	-
trans-β-caryophyllene	1412	1418	0.69	2.8	7.8	2.5	-	-

			V. officinalis		*Hydrocarbon fraction*		*Oxygenated fraction*	
**Compound**	I^t_{Sexp}	I^t_{Slit}	Norm. Rel. % Abundance	RSD%	Norm. Rel. % Abundance	RSD%	Norm. Rel. % Abundance	RSD%
trans-α-Bergamotene	1433	1436	tr	-	0.17	8.8	-	-
α-Humulene	1447	1454	0.07	1.9	0.83	0.1	-	-
Aromadendrene	1454	1461	tr	-	0.07	4.7	-	-
trans-β-farnesene	1457	1458	tr	-	0.22	1.0	-	-
Germacrene D	1475	1480	0.05	0.2	0.51	6.8	-	-
Bicyclogermacrene	1489	1494	0.07	0.7	0.74	6.5	-	-
α-Muurolene	1495	1499	tr	-	0.29	9.3	-	-
β-bisabolene	1505	1509	tr	-	0.30	5.2	-	-
trans-γ-Cadinene	1507	1511	tr	-	0.24	10.8	-	-
cis-δ-Cadinene	1518	1519	0.06	0.8	0.68	1.6	-	-
Caryophyllene oxide	1575	1580	0.07	6.7	-	-	0.10	8.8

Table S5. Normalized percentage abundance of the compounds identified in the EO of Melissa officinalis EO 1 and in its hydrocarbon and oxygenated fractions (tr : trace; - : not detected).

			M. officinalis 1		*Hydrocarbon fraction*		*Oxygenated fraction*	
**Compound**	I^t_{Sexp}	I^t_{Slit}	Norm. Rel. % Abundance	RSD%	Norm. Rel. % Abundance	RSD%	Norm. Rel. % Abundance	RSD%
1-Octen-3-ol	982	978	0.21	4.4	-	-	0.14	10.0
6-Methyl-5-hepten2-one	989	985	1.4	0.50	-	-	0.71	3.1
Linalool	1098	1098	0.32	1.6	-	-	0.28	7.5
Nonal	1103	1098	0.17	1.7	-	-	0.11	3.1
cis-Rose oxide	1109	1111	0.20	0.34	-	-	0.16	8.7
trans-Rose oxide	1126	1127	0.10	0.79	-	-	0.07	10.6
Isopulegol	1143	1146	0.52	2.8	-	-	0.49	4.7
Citronellal	1155	1153	19.6	0.40	-	-	18.3	1.5
Nerol	1229	1229	0.45	3.9	-	-	0.47	7.9
β-Citronellol	1230	1228	4.1	0.72	-	-	4.5	11.0
Neral	1242	1240	19.7	0.08	-	-	22.1	0.9
Piperitone	1252	1252	0.10	2.8	-	-	0.11	6.3
Geraniol	1257	1255	1.7	2.7	-	-	1.83	4.6
Citronellyl acetate	1277		1.6	1.9	-	-	1.6	1.1
Geranial	1273	1270	29.6	0.19	-	-	32.8	1.6
Citronellyl formate	1277	1275	1.0	0.19	-	-	0.14	5.8
Methyl geranate	1324	1323	0.86	1.6	-	-	0.89	5.8
Citronellyl acetate	1355	1354	0.18	4.0	-	-	-	-
Neryl acetate	1366	1365	0.13	35.8	-	-	0.14	1.9
α-Copaene	1371	1371	0.06	0.19	1.84	0.2	-	-
Geranyl acetate	1385	1383	2.5	0.23	-	-	2.8	0.4
β-Elemene	1388	1391	Tr	-	0.96	0.7	-	-
cis-β-Caryophyllene	1399	1404	Tr	-	1.13	0.6	-	-
trans-β-Caryophyllene	1413	1418	2.6	1.4	75.36	0.2	-	-
α-Humulene	1447	1454	0.13	7.0	5.71	0.4	-	-
Aromadendrene	1454	1461	0.07	8.0	2.84	0.6	-	-
α-Muurolene	1494	1499	Tr	-	1.26	1.3	-	-
δ-Cadinene	1518	1524	Tr	-	2.31	1.0	-	-
Caryophyllene oxide	1575	1580	5.7	1.7	-	-	6.3	1.2

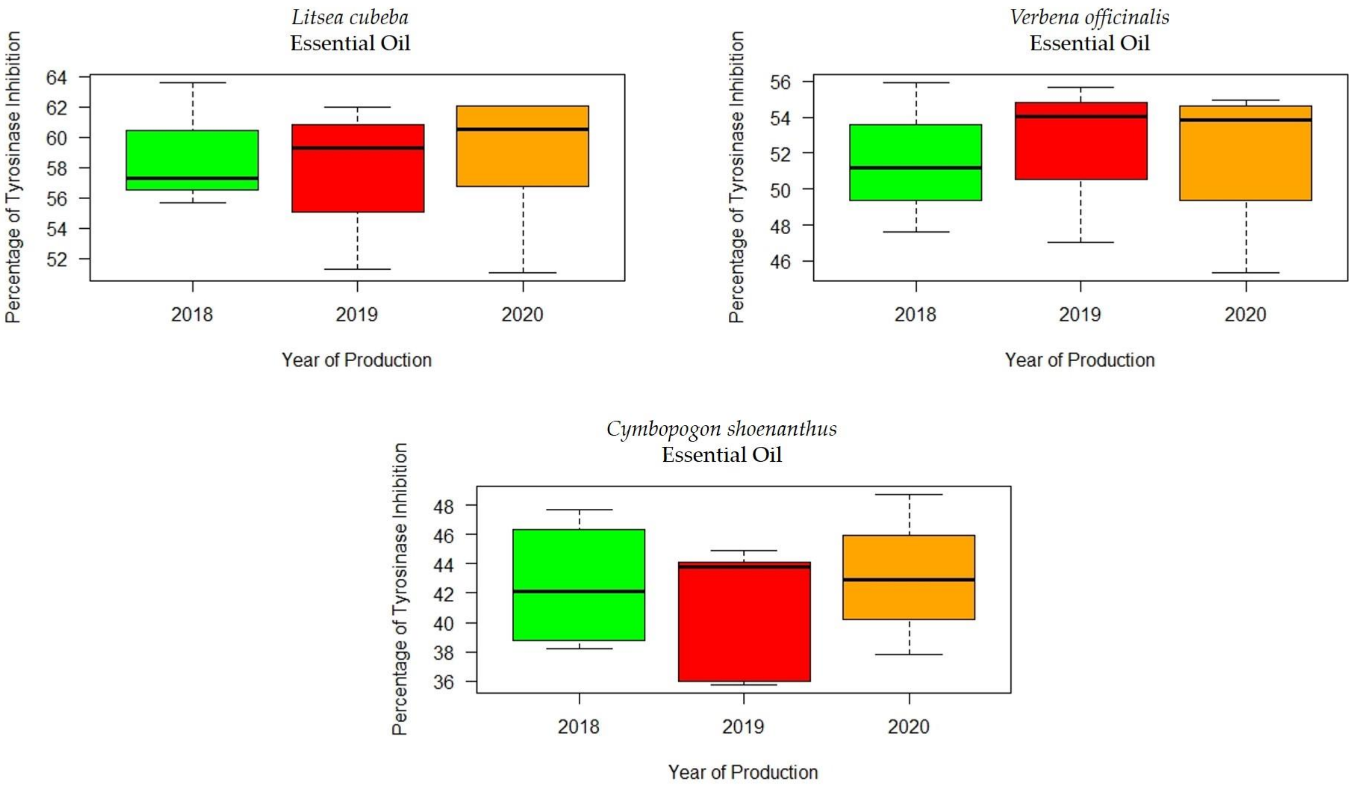

Figure S1. Percentage of tyrosinase inhibition of EOs of different years of production tested at a concentration of 166.7 μg/mL.
